# Effects of Two Workload-Matched High-Intensity Interval Training Protocols on Regional Body Composition and Fat Oxidation in Obese Men

**DOI:** 10.3390/nu13041096

**Published:** 2021-03-27

**Authors:** Spyridon Tsirigkakis, George Mastorakos, Yiannis Koutedakis, Vassilis Mougios, Alan M. Nevill, Zoe Pafili, Gregory C. Bogdanis

**Affiliations:** 1 School of Physical Education & Sports Science, University of Thessaly, 42100 Trikala, Greece; stsirigkakis@uth.gr (S.T.); y.koutedakis@uth.gr (Y.K.); 2 Unit of Metabolism and Endocrinology of Physical Activity and Sport, Department of Medicine, National & Kapodistrian University of Athens, Aretaieion Hospital, 11528 Athens, Greece; mastorakg@gmail.com; 3 Faculty of Education, Health and Wellbeing, University of Wolverhampton, Walsall WS1 3BD, UK; a.m.nevill@wlv.ac.uk; 4 School of Physical Education & Sports Science, Aristotle University of Thessaloniki, 57001 Thessaloniki, Greece; mougios@auth.gr; 5 Department of Dietetics, Evaggelismos General Hospital, 10676 Athens, Greece; zoepafili@gmail.com; 6 School of Physical Education and Sport Science, National and Kapodistrian University of Athens, 17237 Athens, Greece

**Keywords:** obesity, intermittent exercise, weight loss, whole-body fat oxidation

## Abstract

The effects of two high-intensity interval training (HIIT) protocols on regional body composition and fat oxidation in men with obesity were compared using a parallel randomized design. Sixteen inactive males (age, 38.9 ± 7.3 years; body fat, 31.8 ± 3.9%; peak oxygen uptake, VO_2peak_, 30.9 ± 4.1 mL/kg/min; all mean ± SD) were randomly assigned to either HIIT10 (48 × 10 s bouts at 100% of peak power [W_peak_] with 15 s of recovery) or HIIT60 group (8 × 60 s bouts at 100% W_peak_ with 90 s of recovery), and subsequently completed eight weeks of training, while maintaining the same diet. Analyses of variance (ANOVA) showed only a main effect of time (*p* < 0.01) and no group or interaction effects (*p* > 0.05) in the examined parameters. Total and trunk fat mass decreased by 1.81 kg (90%CI: −2.63 to −0.99 kg; *p* = 0.002) and 1.45 kg (90%CI: −1.95 to −0.94 kg; *p* < 0.001), respectively, while leg lean mass increased by 0.86 kg (90%CI: 0.63 to 1.08 kg; *p* < 0.001), following both HIIT protocols. HIIT increased peak fat oxidation (PFO) (from 0.20 ± 0.05 to 0.33 ± 0.08 g/min, *p* = 0.001), as well as fat oxidation over a wide range of submaximal exercise intensities, and shifted PFO to higher intensity (from 33.6 ± 4.6 to 37.6 ± 6.7% VO_2peak_, *p* = 0.039). HIIT, irrespective of protocol, improved VO_2peak_ by 20.0 ± 7.2% (*p* < 0.001), while blood lactate at various submaximal intensities decreased by 20.6% (*p* = 0.001). In conclusion, both HIIT protocols were equally effective in improving regional body composition and fat oxidation during exercise in obese men.

## 1. Introduction

During the last four decades, there has been a dramatic increase in the prevalence of obesity worldwide [1] with trends indicating a further rise until 2030 [2]. Obesity is a disease associated with increased premature morbidity and mortality, which is partially explained by a disturbed energy balance characterized by overfeeding, reduced physical activity and impaired fat oxidation [3]. Endurance exercise is recognized as an important lifestyle intervention in weight management, primarily by creating an energy deficit in a dose–response relationship with training volume [4]. However, at least one out of four adults does not meet the physical activity guidelines of 150 min of moderate-to-vigorous physical activity per week [5]. Among the main barriers to physical activity are the reported lack of time and the inability to adhere to lengthy exercise programs [6].

High-intensity interval training (HIIT) has been shown to be an effective and time-efficient form of aerobic training [6]. Accumulating evidence suggests that HIIT can act as a “gateway to exercise”, helping individuals to initiate an exercise program, as it reduces the time commitment to exercise, while having positive effects on body composition and energy metabolism [7].

A limited number of studies on sedentary overweight/obese individuals have shown an increase in fat oxidation rates both at rest and during exercise after HIIT, but findings regarding changes in body composition are conflicting [8,9]. An increase in fat oxidation and a possible change in body composition following HIIT may be more important in obese individuals, who usually demonstrate low fat oxidation rates during both rest and exercise [10]. The majority of interventional HIIT studies have examined whole-body fat oxidation during a single moderate-intensity bout of exercise (e.g., 65% of peak oxygen uptake, VO_2peak_) [11,12], despite the fact that the intensity at which maximal fat oxidation is achieved differs among individuals, occurring between 45 and 65% VO_2peak_ in healthy adults [13]. Importantly, in sedentary overweight individuals, peak fat oxidation (PFO) may occur at lower intensities (~40% VO_2peak_) [14]. Given that fat oxidation capacity is strongly associated with metabolic health [15], it is important to examine how this parameter is affected by HIIT in overweight/obese populations during exercise at a wide range of intensities.

Augmented abdominal fat deposition is a strong predictor of cardiometabolic risk, independently of total body fat [16], due to its increased metabolic activity and anatomical position near to the portal vein [17]. Visceral fat is related with cardiometabolic risk factors, such as high blood pressure, high levels of triglycerides, high low-density lipoprotein cholesterol, and abnormal glucose metabolism [16,17]. Recent studies have indicated that HIIT may reduce total body and trunk fat levels in inactive individuals of normal weight [18] and in overweight persons [19]. However, there is a gap in the literature regarding the effectiveness of different HIIT protocols in reducing total and regional adiposity. A previous study demonstrated that a 12-week HIIT intervention in obese young women, using 4 min bouts of cycling at 90% VO_2peak_, resulted in similar significant reductions of total body mass and fat mass compared to sprint interval training of equal workload [20]. Nevertheless, HIIT induced greater trunk fat loss compared to sprint interval training, indicating that exercise format could be a decisive factor for regional fat loss. This may be due to differences in the acute metabolic responses of different exercise protocols despite a similar total energy expenditure [21], possibly inducing different adaptations [22]. Previous studies have shown that, in workload-matched protocols of high-intensity intermittent exercise, bout duration has a large impact on physiological and metabolic responses [23,24,25]. For example, during repeated bouts of intense exercise (120% VO_2peak_) executed for 40 min, blood lactate was 50–60% higher and fat oxidation rate was 50% lower when bout duration was 24 s compared with 6 s, despite a similar total workload [23,24]. This may imply that shorter bouts of HIIT may have a different effect on fat metabolism and body composition. However, the training-induced changes in fat metabolism and regional body composition following different HIIT protocols in overweight/obese adults are still unknown. The purpose of the present study was to compare the effects of bout duration (10 and 60 s) during two HIIT programs, matched for workload, on regional body composition, and fat oxidation during exercise, in obese adult males. It was hypothesized that the protocol using shorter bout duration would result in greater improvements in fat oxidation and body fat loss after training, despite the equal workload of the two HIIT protocols.

## 2. Materials and Methods

### 2.1. Participants

Power analysis indicated that a sample size of 7 participants per group would be needed to detect significant differences if the effect size (ES) was 0.3. Power analysis was performed using the following additional parameters: type of analysis was set to repeated-measures within-between interaction ANOVA, the required power was set to 0.80, alpha was set to 0.05, and the correlation coefficient between repeated measures was set to 0.5 (G-Power software, v. 3.1.9.2, Universität Kiel, Kiel, Germany.

Participants were recruited via advertisements placed at affiliated University hospitals of the Medical School of Athens, electronic bulletins, and word of mouth. All volunteers completed a comprehensive medical history and a validated physical activity [26] questionnaire. Inclusion criteria were (a) healthy males aged 18–50 years, (b) body mass index (BMI) of 28–35 kg/m^2^ and percent fat mass (%FM) ≥ 25%, (c) low-to-moderate physical activity and no participation in any structured exercise programs during the 12 month period before entering the study. Exclusion criteria were (a) use of tobacco and related products in the past 6 months, (b) weight change > 2 kg during the 6 months preceding the study, (c) participation in a weight loss program over the 6 months preceding the study, (d) use of dietary supplements and medications during the 6 months preceding the study, (e) history of endocrine or metabolic disorders as well as chronic diseases or family history of early cardiac mortality and diabetes, and (f) depression or other mental disorders.

**Figure 1:**
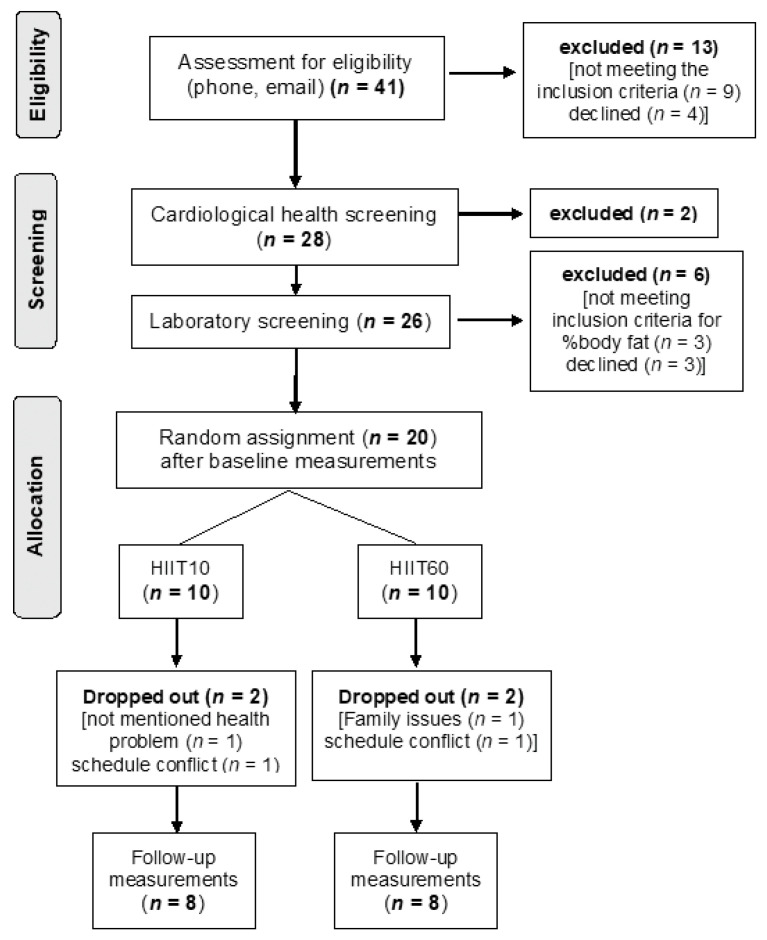
Flow diagram of the study design.

A total of 41 individuals volunteered and were assessed for eligibility between September 2017 and May 2018 (Figure 1). Of these, 20 were eligible and were assigned to the two intervention groups; 16 completed the study. Demographic characteristics of the two groups were similar at the start of the intervention (age: 37.2 ± 9.5 vs. 40.2 ± 3.9 years, *p* = 0.369; height: 176 ± 6 vs. 177 ± 9 cm, *p* = 0.792, body mass: 91.94 ± 7.92 vs. 94.26 ± 14.10 kg, *p* = 0.691, BMI: 29.8 ± 2.1 vs. 30.1 ± 2.6 kg/m^2^, *p* = 0.805 for HIIT10 and HIIT60, respectively). Furthermore, the baseline values of the VO_2peak_ test (Table 2), total and regional body composition (Table 3), and substrate oxidation (Table 4) were similar in the two groups (*p* > 0.05).

After a thorough explanation of the testing protocol, the possible risks involved and the right to withdraw at will, written informed consent was obtained from each participant. Ethical approval was obtained by the Aretaieion Hospital Research Ethics Committee (B−153/4–2–2016), and all procedures were in accordance with the Code of Ethics of the World Medical Association (Helsinki Declaration of 1964, as revised in 2013).

### 2.2. Study Overview

A parallel randomized design was used to compare the effects of bout duration (10 and 60 s) during two HIIT programs, matched for workload, on regional body composition and fat oxidation during exercise in obese adult males. A flow diagram of the study is presented in Figure 1. The timeline of the study included: (1) medical clearance and familiarization procedures, (2) baseline (pre) testing, (3) an 8-week HIIT intervention, and (4) post-training testing. Following baseline testing, subjects were randomly divided into two groups: HIIT10 (*n* = 8) and HIIT60 (*n* = 8). A block randomization, with the aid of a custom-made computer routine, was used to equally allocate participants in the two groups without any stratification factor. Both groups performed 24 cycling interval training sessions (3 sessions, separated by at least 48 h, per week for 8 weeks) of equal total duration (20 min), mechanical work, with work-to-recovery time ratio (1:1.5). Specifically, total exercise time was 8 min, total recovery time was 12 min and total session duration, including warm-up, was 23 min. Participants in the HIIT10 group performed 48 repetitions of 10 s cycling at 100% peak power (W_peak_), with 15 s active recovery at 15% W_peak_; participants in the HIIT60 protocol performed 8 repetitions of 60 s each, with 90 s active recovery at the same intensities. At the end of the fourth week of the intervention, W_peak_ was measured to readjust the workload.

The dependent variables were: VO_2peak_, peak heart rate (HR_peak_), W_peak_, blood lactate, body mass, BMI, waist circumference, total and regional body fat and lean mass, fat oxidation rates at 6 submaximal intensities, PFO, the relative exercise intensities corresponding to PFO, as well as fat and carbohydrate crossover point (i.e., the intensity of exercise at which energy derived from oxidation of carbohydrate equals that derived from fat).

All assessments were performed at the exercise physiology laboratory of the Unit of Metabolism and Endocrinology of Physical Activity and Sport and at the department of radiology of the Medical School of the National and Kapodistrian University of Athens.

### 2.3. Anthropometry and Body Composition

On the first of three preliminary visits, standing height was measured to the nearest 0.1 cm (Seca 213, Hamburg, Germany). Furthermore, body mass was measured to the nearest 0.1 kg (Seca 888, Hamburg, Germany), with participants wearing only underwear. Waist circumference measurements were also performed with a non-elastic tape (Seca 203, Hamburg, Germany) to the nearest 0.1 cm by the same experienced researcher before and after the intervention, according to standardized procedures [27].

Body composition, including total and regional fat mass (FM), fat-free mass (FFM) and bone mineral content (BMC), were measured by whole-body dual-energy X-ray absorptiometry (DEXA) (Lunar Prodigy, GE Healthcare, Madison, WI, USA). Analysis was performed using the Lunar enCORE software version 11 according to standard procedures, as previously described [28]. The DEXA instrument was calibrated each day before measurements according to the manufacturer’s instructions using a QA block phantom. All measurements were carried out in the morning (8:00–10:00) after an overnight fast. All DEXA scans were performed and analysed by the same certified technician. Anthropometric and body composition measurements were repeated 72 to 96 h after the final HIIT session.

### 2.4. Peak Oxygen Uptake and Substrate Oxidation during Submaximal Cycling

On the second visit, VO_2peak_ and W_peak_ were determined by a continuous graded exercise protocol lasting 8–12 min on the same electronic cycle ergometer (ergo bike premium 8i DAUM, Germany) as that used in training, in controlled environmental conditions (ambient temperature, 20–23 °C; relative humidity, 45–55%). The cycle ergometer controlled the resistance, so that the pre-set power output was maintained, despite small fluctuations in cadence. Participants were instructed to pedal at a constant frequency of 70 rpm, directed by a metronome and visual inspection of the ergometer panel. The protocol began at 20–35 W and power was increased by 20–25 W per minute until volitional fatigue. The initial power and the workload increment per stage were determined during familiarization, from the individual linear regression between heart rate and power at 4 different workloads, taking into account the predicted peak HR and a desired test duration of 10 min [29]. Verbal encouragement was given during the last part of the test. All participants achieved VO_2peak_ according to standard ACSM criteria [30]. Minute ventilation (VE), oxygen uptake (VO_2_), and carbon dioxide output (VCO_2_), were collected breath by breath using a metabolic cart (MGC Diagnostics, Ultima CPX™, Saint Paul, MN, USA), which was calibrated before each trial according to the manufacturer’s procedures. Data were averaged every 10 s, and VO_2peak_ was calculated as the mean of the three highest consecutive 10 s values. W_peak_ was calculated as the power of the last completed stage. Heart rate (HR) was monitored continuously during exercise by a HR monitor (Polar S810i, Polar Electro Inc. Lake Success, NY, USA), and the highest value was taken as HR_peak_. The graded exercise protocol to determine VO_2peak_ and W_peak_ was repeated at the end of the fourth week of training and 96–120 h after the final HIIT session (i.e., 24–48 h after the DEXA scan).

On the third preliminary visit, participants performed a submaximal cycling test to calculate substrate utilization. The protocol consisted of six 4 min continuous workloads at 15%, 30%, 40%, 50%, 60% and 75% of baseline W_peak_. Non-protein substrate oxidation rates, expressed in absolute values (g/min and kcal/min), were calculated using stoichiometric equations from VCO_2_ and VO_2_, measured at steady state during the last minute of each stage [31,32]. From these data, PFO rate and the corresponding exercise intensity at which it was achieved (Fat_peak_) were calculated using a third-degree polynomial fit. Also, the crossover point, i.e., the exercise intensity at which energy derived from oxidation of carbohydrate was equal to the energy derived from fat, was calculated. The highest value of carbohydrate oxidation calculated from the stoichiometric equations was termed the peak carbohydrate oxidation rate (PCHO). Capillary blood samples were collected during the last 30 s of each stage from a fingertip for lactate determination via a portable device (Lactate Scout^+^; EKF, Barleben, Germany), and HR was recorded every 5 s with the aforementioned HR monitor. The submaximal test was performed again after the end of the HIIT, and at least 48 h after the VO_2peak_ test.

### 2.5. Training Protocols

The two groups trained 3 times per week for 8 weeks. Each session lasted 20 min, and sessions were separated by at least 48 h. Each participant performed all the training sessions at a specific day time. Training was standardized for time of day for each participant (±2 h) and all training sessions took place in a temperature-controlled laboratory (20–21 °C), under the supervision of experienced research staff.

In each training session, the HIIT10 group performed 48 × 10 s repetitions at a steady cadence (70 rpm). Exercise intensity was set to 100% of W_peak_, alternating with 15 s active recovery at 15% W_peak_. The HIIT60 group performed 8 × 60 s repetitions at 100% of W_peak_ (70 rpm), with 90 s active recovery at 15% W_peak_. At the end of the fourth week of the intervention, exercise intensity was readjusted based on a mid-intervention maximal test, the same as that used initially. All training sessions were performed in the morning (between 8:00 and 10:00). Each session started with 5 min of warm-up at 15% W_peak_ and was followed by 3 min of cool down. HR was recorded continuously using the aforementioned monitor during all training sessions. Total energy expenditure during the training session was calculated via indirect calorimetry during the first and last (24th) training session [33].

### 2.6. Dietary Intake and Habitual Physical Activity Assessment

Participants recorded their food and drink intakes for two days prior to the first exercise test and were requested to consume the same food types and quantities before each subsequent test. Furthermore, participants were required to maintain their daily dietary intake and physical activity habits and patterns throughout the intervention period. Detailed, 3-day records (2 weekdays and 1 weekend day) of weighed food and beverage intake were collected from each participant at baseline, at mid-intervention (4th week) and during the last week of the training intervention. Energy intake and diet composition were analysed by an experienced dietitian using appropriate software (Axxya Systems Nutritionist Pro TM 2011). Participants also completed the Greek version of the long form International Physical Activity Questionnaire (IPAQ) during an interview with the researchers and wore a pedometer (Tanita PD-637, Tokyo, Japan) for one week. IPAQ and pedometer data refer to the first and eighth (last) week of training.

### 2.7. Statistical Analysis

The Shapiro–Wilk, Levene and Mauchly’s tests were used to verify the normality, homogeneity and sphericity of the sample data, respectively. Three-way, mixed-factor analyses of covariance (ANCOVA; 2 training groups × 2 time-points × 6 stage measurements) were conducted to examine differences in fat oxidation rates and blood lactate measured during the submaximal test, using power output as a covariate. To further examine differences in the responses to HIIT between the two groups at each one of the six submaximal exercise stages, follow-up 2-way ANCOVA (group × time) was used for each stage separately, using power output as a covariate.

Differences in VO_2peak_, W_peak_, regional and total body composition, anthropometric variables, PFO rate, crossover point, Fat_peak_, daily energy intake, weekly energy expenditure (IPAQ) and number of daily steps were evaluated using a 2-way mixed-factor ANOVA (2 training groups × 2 time points). Significant main effects or interactions were further examined by Tukey’s post hoc tests. Effect sizes (ES) for AN(C)OVA were determined by partial eta squared (η^2^) and were declared as small (0.01 to 0.059), moderate (0.06 to 0.137), or large (>0.138) according to Cohen [34]. For pairwise comparisons, ES was determined by Hedge’s g (small: 0.2–0.5, moderate: 0.5–0.8 and large: >0.8). Analyses were performed using the IBM SPSS Statistics for Windows, Version 23.0 (Armonk, NY: IBM Corp). All data are presented as mean ± standard deviation (SD). Significance was set at *p* < 0.05.

## 3. Results

### 3.1. Energy Intake and Habitual Physical Activity

For daily energy intake, the 2-way ANOVA showed no significant main effect for group, time or group × time interaction (Table 1). There were also no changes in the macronutrient composition of the diet throughout the intervention (Table 1). Analysis of the weekly energy expenditure, as assessed via IPAQ, showed no significant main effect for group (*p* = 0.827), time (*p* = 0.761) or group × time interaction (*p* = 0.499). Weekly energy expenditure in the first and last week of the intervention period was: HIIT10, 2314 ± 987 vs. 2275 ± 914 MET min; HIIT60, 2350 ± 1000 vs. 2452 ± 1022 MET min. Likewise, the 2-way ANOVA for the number of daily steps showed no significant main effect for group (*p* = 0.950), time (*p* = 0.292) or group × time interaction (*p* = 0.679). The number of daily steps in weeks 1, 4 and 8 were, respectively, as follows: HIIT10, 5511 ± 2206; 5287 ± 2141; 5595 ± 2157 and HIIT60, 5393 ± 216; 5350 ±2120; 5447 ± 1992).

### 3.2. Exercise Compliance

Attendance of training sessions during intervention was 95 ± 4% and 94 ± 5% in HIIT10 and HIIT60, respectively. No physical injuries and no adverse events were reported during the experimental period in either group.

### 3.3. Power Output and Energy Expenditure during HIIT Sessions

For mean power during the trials, the 2-way ANOVA revealed no significant main effect of group (*p* = 0.164) or time × group interaction (*p* = 0.738). Mean power values increased significantly in both groups from the first to the last training session (HIIT10, 95 ± 9 vs. 113 ± 4 W; HIIT60, 105 ± 16 W vs. 122 ± 19 W, *p* < 0.001). Similarly, no significant effect of group (*p* = 0.209) or time × group interaction (0.543) was found for energy expenditure during the trials. Nevertheless, the average energy expenditure, as assessed by indirect calorimetry, in the first and the 24th training session was: HIIT10, 199 ± 25 vs. 276 ± 21 kcal; HIIT60, 234 ± 53 vs. 300 ± 74 kcal, *p* < 0.001).

### 3.4. Cardiorespiratory Fitness

No significant group × time interaction or main effect of group was found in VO_2peak_ and W**_peak_** (Table 2). However, there was a significant main effect of time, showing large improvement of VO_2peak_ (by 20.0 ± 7.2%, *p* < 0.001, ES = 1.42) and W**_peak_** (by 18.3 ± 6.6%, *p* < 0.001, ES = 1.28) after training for both groups (Table 2).

### 3.5. Anthropometric and Body Composition Variables

There was no significant group × time interaction or main effect of group on any anthropometric or body composition variable (Table 3). However, there was a main effect of time on body fat, showing a decrease in total and segmental fat mass in both groups. The decrease in total fat mass was attributable mainly to loss of trunk fat mass (1.45 of 1.81 kg of total fat mass loss). The latter was accompanied by a decrease in waist circumference (Table 3). Moreover, there was an increase in lean body mass of 0.82 ± 0.55 kg, which was attributable mainly to the increase in leg lean body mass, as there were minimal, non-significant changes in arm and trunk lean mass, and total body bone mineral content (Table 3).

### 3.6. Substrate Oxidation Indices and Blood Lactate Concentration during Submaximal Exercise

**Figure 2:**
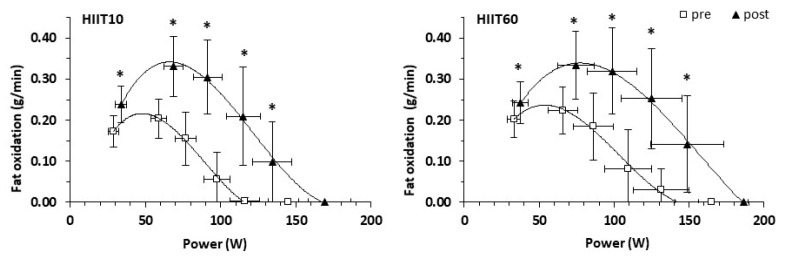
Fat oxidation rate during a progressive submaximal exercise test before (pre) and after (post) training for the HIIT10 and the HIIT60 groups. * *p* < 0.001 for pre- vs. post-training. Points represent means, and error bars represent standard deviation.

For fat oxidation rate during the six submaximal exercise stages, the 3-way ANCOVA revealed a main effect of time (*p* < 0.001) and stage (*p* < 0.001), as well as a group × stages × time interaction (*p* = 0.003). The follow-up 2-way ANCOVA performed for each submaximal stage showed that there were no group × time interactions (*p* = 0.427 to 0.971) or main effects of group (*p* = 0.492 to 0.943) for fat oxidation during any of the submaximal test stages (Figure 2). However, there was a main effect of time for the first five stages (*p* = 0.001 to 0.002), but not for the last stage (*p* = 0.234). Specifically, fat oxidation increased at the 1st stage by 0.05 g/min (*p* < 0.001, ES = 1.15), at the 2nd stage by 0.12 g/min (*p* < 0.001, ES = 1.61), at the 3rd stage (by 0.14 g/min, *p* < 0.001, ES = 1.64), at the 4th stage (by 0.16 g/min; *p* < 0.001, ES = 1.56) and at the 5th stage (by 0.10 g/min; *p* = 0.002, ES = 1.56). The average overall increase in fat oxidation after training was 88.6% (from 0.11 ± 0.10 to 0.21 ± 0.14 g/min, *p* < 0.001, ES = 0.77).

Moreover, training resulted in a significant increase in PFO, expressed in absolute terms (by 60.0 ± 46.8%) with no significant difference between groups (Table 4). The relative exercise intensity corresponding to PFO, i.e., Fat_peak_, expressed as a percentage of VO_2peak_ or percentage of HR_peak_, increased similarly in both groups (from 33.6 ± 4.6 to 37.6 ± 6.7% VO_2peak_; main effect of time: *p* = 0.039, and from 59.4 ± 6.4 to 62.9 ± 4.8% HR_peak_; main effect of time: *p* = 0.011, Table 4). No main effect of group or group × time interaction was found for the crossover point (Table 4). However, there was a main effect of time, and following HIIT, participants shifted their crossover point to higher relative intensities (from 31.1 ± 6.1 to 38.8 ± 8.7% VO_2peak_; main effect of time: *p* = 0.023, Table 4). There was no main effect of group or group × time interaction for PCHO (Table 4). However, PCHO increased similarly in both groups after training (Table 4).

**Figure 3:**
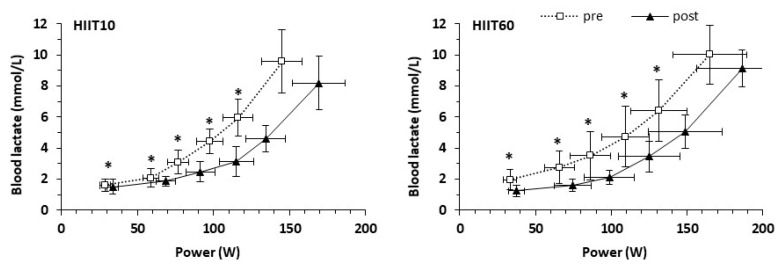
Blood lactate concentration during a progressive submaximal test before (pre) and after (post) training for the HIIT10 and the HIIT60 groups. * *p* < 0.05 for pre- vs. post-training. Points represent means, and error bars represent SD. VO_2_, oxygen uptake.

Blood lactate concentration in the two groups during the six submaximal exercise stages, before and after training, is shown in Figure 3. The 3-way ANCOVA for blood lactate concentration during the six submaximal exercise stages revealed a main effect of time (*p* < 0.001) and stage (*p* < 0.001), but not a group × stage × time interaction (*p* = 0.819). The follow-up 2-way ANCOVA performed for each submaximal stage showed that there were no group × time interactions (*p* = 0.090 to 1.000) or main effects of group (*p* = 0.397 to 0.897) for blood lactate concentration during any of the submaximal test stages (Figure 3). However, there was a main effect of time for the first five stages (*p* = 0.017 to 0.033), but not for the last stage (*p* = 0.087). The average overall decrease of blood lactate concentration after training was 20.6% (*p* = 0.001, ES = 0.33).

## 4. Discussion

The main finding of the study was that in obese males, eight weeks of training using two HIIT programs with different bout duration (10 and 60 s) but equal workload and intensity were equally effective in inducing beneficial changes in regional body composition and whole-body fat oxidation, without any changes in dietary energy intake. The results of this study highlighted that regular physical exercise and diet are both important for effective obesity prevention and management, since “intake” and “expenditure” are the two sides of the same coin [3]. Additionally, both HIIT programs resulted in significant improvements in VO_2peak_ and in blood lactate concentration during submaximal exercise.

In recent years, HIIT has been a major trend in the fitness industry [35], as numerous studies have shown that HIIT is a time-efficient model of exercise to improve fitness and cardiometabolic health [36,37]. Nevertheless, investigation of the effectiveness of different HIIT programs to combat obesity has not received adequate attention. In the present study, two isoenergetic HIIT protocols, differing in bout duration, were equally effective in reducing trunk body fat by 1.45 kg and waist circumference by about 3%. Similar reductions in trunk fat mass have been recently reported following 12 weeks of HIIT and sprint interval training of equal external work rate [38]. One possible explanation for the reduction in body fat and the lack of differences between the two HIIT protocols may be energy deficit. Considering that energy expenditure per session was similar in the two protocols (around 250 kcal), it may be calculated that, on average, a total of 6000 kcal were expended during all training sessions. This energy expenditure may account for the largest part of body mass loss over the 8 weeks of training, taking into consideration that no significant changes in energy and macronutrient intake or habitual physical activity were reported during the study period.

Another mechanism that may contribute to the reduction in body fat following 8 weeks of HIIT is increased post-exercise energy consumption, which is observed for a few h after intense exercise [39]. Several studies have demonstrated that different forms of high-intensity intermittent exercise result in elevated energy expenditure and altered substrate oxidation over the 24 h following exercise [21,40,41]. This energy may be used for glycogen resynthesis from lactate [42], hormone balancing, cellular repair and anabolic processes [43]. Thus, the large metabolic disturbances and muscle load due to HIIT may result in an additional 100–160 kcal of energy per session being expended during the 24 h of recovery [44].

In the present study, both HIIT10 and HIIT 60 elicited a 0.86 kg gain in muscle mass of the legs, which may cause an increase in resting metabolic rate [45]. An increase in leg muscle mass following HIIT has been previously observed in overweight subjects [8], mainly following cycling exercise. This local muscle hypertrophy may be caused by the high forces exerted by the leg muscles during HIIT on a cycle ergometer, rendering cycling an effective, and at the same time a safe, way to increase muscle mass in overweight/obese individuals. Furthermore, activation of inflammatory processes due to HIIT leads to enhanced muscle protein turnover, which not only consumes energy, but also leads to muscle hypertrophy in response to training [46]. Increased muscle mass is related to elevated resting metabolic rate, which represents the largest proportion of total daily expenditure in sedentary individuals [47]. A higher resting metabolic rate due to increased muscle mass creates a constant “energy deficit”, leading to weight loss, and may help to promote body fat reduction due to training [48]. Thus, the summed energy deficit created by HIIT and the associated post-exercise energy expenditure, as well as the possibly increased basal metabolic rate due to increased muscle mass, may explain the body mass and fat mass loss observed in the present study, taking into account that energy intake was unaltered throughout the duration of the intervention.

Another main finding of the present study was the considerable increase in fat oxidation over a wide range of exercise intensities (≈20–80% VO_2peak_), including a large increase in PFO, which was shifted to higher exercise intensity with HIIT. An increase in fat oxidation due to HIIT may contribute to fat loss, as impaired skeletal fat oxidation has an important contribution to the development and maintenance of obesity [10]. Thus, a shift of substrate oxidation during exercise following HIIT training may be a contributing factor to the observed fat loss. A previous study using HIIT in sedentary women has shown significant changes in visceral adipose tissue coupled with increased fat oxidation during very-low-intensity exercise [49]. This finding is in accordance with our results, which show that improved fat oxidation is observed over a wide range of intensities and not only at a fixed intensity or a peak value. The combination of reduced fat, especially from the abdominal area, and increased oxidative capacity may also improve cardiometabolic health [50]. Other studies have shown that improved fat oxidation during exercise is linked with improved insulin sensitivity following 6 weeks of sprint interval training (6 × 6 s of supramaximal cycling with 2 min rest) in previously sedentary obese young adults of both sexes [9]. In contrast, 12 weeks of lower-intensity interval training (5 × 2 min at an intensity reaching 95% HR_max_) was less efficient than prolonged running (60 min at 80% HR_max_) in lowering fat percentage or enhancing fat oxidation in untrained overweight young men [51]. This may indicate that energy expenditure during exercise may be an important factor for body composition and fat oxidation changes. Notably, when training with short sprints (80 × 6 s “all-out” cycling sprints with rest intervals of 9 s) was compared with HIIT (repeated 4 min bouts at 90% VO_2max_ with 3 min of passive recovery) of equal workload (400 kJ), there were similar reductions in trunk and total fat mass in active obese female students [20]. This may explain why, in the present study, changes in body composition and fat oxidation were not affected by HIIT format (bouts of 10 or 60 s), possibly due to the equal energy expenditure and total workload.

The increased fat oxidation during submaximal exercise after HIIT may be explained by metabolic adaptations at the level of skeletal muscle and improvements in cardiorespiratory capacity, as reflected by increased VO_2peak_ [6]. In the present study, VO_2peak_ increased by 20.0%, which is in accordance with previous findings of studies using different protocols of interval training in inactive overweight men [52] and untrained subjects [53]. The large improvement in maximal exercise performance could be due to the low initial level of VO_2peak_ of the participants [54] and to the effectiveness of the protocols used. Notably, a study using 60 bouts of cycling for 8 s at ∼90% of VO_2peak_ with 12 s of passive recovery in between showed an improvement in VO_2peak_ of only 7.3% after 20 training sessions in overweight/obese young women [55]. In another study where healthy middle-aged obese subjects trained for 6 weeks using cycling interval training (7 × 1 min at 100% of VO_2peak_ with 1 min of passive recovery in between), a 9% improvement in VO_2peak_ was observed [56]. A possibly increased higher oxidative capacity after training may contribute to higher fat oxidation and fat loss [50]. It has been found that a high-volume HIIT resulted in improvements in oxidative capacity at the whole-body level during 1 h cycling at 60% of pre-training VO_2peak_ in recreationally active individuals [11]. The key mechanism underlying these metabolic adaptations to different forms of HIIT is enhanced skeletal muscle mitochondrial content [57]. Previous studies have shown strong correlations between PFO and skeletal muscle oxidative capacity, reflected by increased muscle deoxygenation kinetics [58], and greater proportion of type I muscle fibers [59]. In the present study, blood lactate responses to submaximal exercise were significantly reduced (by about 20%), suggesting improved muscle oxidative capacity [60]. Blood lactate levels and the corresponding [H^+^] may also modulate fat oxidation, as lactate levels and [H^+^] influence plasma-free fatty acid concentration [12]. Thus, a decrease in blood lactate (and H^+^) responses may facilitate free fatty acid mobilization and may partly explain the increased fat oxidation following 8 weeks of HIIT.

The present study has certain limitations, such as the absence of a control, non-training, group. However, there is no scientific basis to assume that any of the dependent variables assessed in this study could change after eight weeks of no training. Thus, it was not deemed acceptable to subject overweight/obese individuals to the demanding tests of the study twice in eight weeks, with no intervention in between. Another limitation may be the fact that diet was recorded at the start of the intervention, and it is unknown whether the participants modified their diet due to their participation in the study. However, the fact that dietary habits and energy intake remained unchanged throughout the 8 weeks of intervention indicates that diet modification did not affect the outcomes of the study.

## 5. Conclusions

Both HIIT protocols were equally effective in inducing a significant reduction in trunk fat, and an increase in leg muscle mass and fat oxidation, while maintaining the same diet and energy intake in obese men. Bout duration did not modify these responses, possibly due to the equal workload and energy expenditure. The findings of the present study provide evidence that changes in body composition and fat oxidation are evident in obese men after 8 weeks of training with either protocol. Based on these results, practitioners may apply these cycling HIIT programs interchangeably in obese men as a safe and effective way to increase leg muscle mass, decrease trunk fat mass and elevate fat oxidation during exercise. 

## Figures and Tables

**Table 1:** Energy and macronutrient intake at the start (week 1), middle (week 4) and last week (week 8) of the intervention for the two training groups (HIIT10 and HIIT60).

		HIIT10			HIIT60			
	Week 1	Week 4	Week 8	Week 1	Week 4	Week 8	Effects	*p*-Value
**Energy**	1985	1707	1852	2313	2280	2327	Time	0.480
**intake**	±514	±441	±524	±796	±707	±795	Group	0.132
**(kcal)**							Interaction	0.632
**CHO**	39.8	42.5	42.2	43.4	40.3	41.5	Time	0.990
**(%)**	±10.4	±11.5	±12.3	±5.6	±3.9	±7.4	Group	0.947
							Interaction	0.600
**Fat**	41.2	38.7	40.0	40.0	40.0	40.2	Time	0.921
**(%)**	±9.7	±11.7	±10.5	±5.9	±8.6	±11.3	Group	0.975
							Interaction	0.914
**Protein**	19.0	18.7	17.9	16.7	19.6	18.3	Time	0.562
**(%)**	±5.2	±3.8	±3.3	±3.8	±6.9	±5.5	Group	0.869
							Interaction	0.436

Values are expressed as mean ± standard deviation; CHO: carbohydrate.

**Table 2:** Peak values of oxygen uptake, heart rate and power before (pre) and after (post) intervention in the two training groups (HIIT10 and HIIT60).

	HIIT10		HΙΤ60			Pre vs. Post		
	Pre	Post	Pre	Post	Pre vs. Post Main Effect	Change (90 CI%); Hedges g	Group	Interaction
**VO_2peak_ (mL/kg/min)**	29.4 ± 2.4	36.0 ± 2.1	32.5 ± 5.0	38.1 ± 5.5	***p* < 0.001, η^2^ = 0.495**	6.1 (5.3, 6.8), ES = 1.42	*p* = 0.534, η^2^ = 0.028	*p* = 0.233, η^2^ = 0.104
**HR_peak_ (beats/min))**	175 ± 10	177 ± 7	177 ± 9	173 ± 8	***p* = 0.718, η^2^ = 0.010**	−1 (−5, 3), ES = 0.11	*p* = 0.817, η^2^ = 0.004	*p* = 0.248, η^2^ = 0.094
**W_peak_ (W)**	193 ± 16	231 ± 9	214 ± 32	250 ± 40	***p* < 0.001, η^2^ = 0.905**	37 (32, 42), ES = 1.28	*p* = 0.152, η^2^ = 0.141	*p* = 0.141, η^2^ = 0.007

Values are means ± standard deviation. Bold font highlights statistically significant differences. 90% CI, 90% confidence interval; η^2^, partial eta squared; ES, effect size; HR_peak_, peak heart rate; VO_2peak_, peak oxygen uptake; W_peak_, peak power.

**Table 3:** Anthropometric and body composition variables before (pre) an after (post) the intervention in the two training groups (HIIT10 and HIIT60).

	HIIT10		HΙΤ60			Pre vs. Post		
	Pre	Post	Pre	Post	Pre vs. Post Main Effect	Change (90 CI%); Hedges g	Group	Interaction
**Body mass (kg)**	91.94 ± 7.92	90.17 ± 7.20	94.26 ± 14.10	93.88 ± 14.41	*p* = 0.043, η^2^ = 0.262	−1.12 (−1.88, −0.35), ES = 0.09	*p* = 0.765, η^2^ = 0.007	*p* = 0.175, η^2^ = 0.127
**BMI (kg/m^2^)**	29.8 ± 2.1	29.2 ± 1.8	30.1 ± 2.6	30.0 ± 2.7	*p* = 0.037, η^2^ = 0.274	−0.36 (−0.62, −0.10), ES = 0.16	*p* = 0.655, η^2^ = 0.015	*p* = 0.166, η^2^ = 0.133
**WC (cm)**	103.7 ± 7.3	99.9 ± 7.0	102.4 ± 9.6	100.7 ± 9.8	*p* < 0.001, η^2^ = 0.626	−2.8 (−3.8, −1.8), ES = 1.64	*p* = 0.952, η^2^ = 0.000	*p* = 0.077, η^2^ = 0.206
**Total body fat (%)**	31.5 ± 4.0	29.4 ± 4.2	32.1 ± 3.9	31.0 ± 3.1	*p* = 0.043, η^2^ = 0.262	−1.6 (−2.7, −0.4), ES = 0.41	*p* = 0.577, η^2^ = 0.023	*p* = 0.514, η^2^ = 0.031
**Total fat mass (kg)**	29.12 ± 5.68	26.68 ± 5.35	30.26 ± 6.15	29.07 ± 5.30	*p* = 0.002, η^2^ = 0.495	−1.81 (−2.63, −0.99), ES = 0.32	*p* = 0.534, η^2^ = 0.028	*p* = 0.233, η^2^ = 0.104
**Trunk fat mass (kg)**	17.64 ± 3.06	15.87 ± 2.78	17.34 ± 3.49	16.21 ± 3.26	*p* < 0.001, η^2^ = 0.612	−1.45 (−1.95, −0.94), ES = 0.46	*p* = 0.991, η^2^ = 0.000	*p* = 0.321, η^2^ = 0.070
**Leg fat mass (kg)**	8.86 ± 2.49	8.42 ± 2.37	9.77 ± 3.12	9.81 ± 2.48	*p* = 0.235, η^2^ = 0.099	−0.20 (−0.48, 0.07), ES = 0.08	*p* = 0.393, η^2^ = 0.053	*p* = 0.174, η^2^ = 0.128
**Arm fat mass (kg)**	2.63 ± 0.80	2.39 ± 0.71	3.15 ± 0.89	3.06 ± 0.90	*p* = 0.077, η^2^ = 0.206	−0.17 (−0.31, −0.02), ES = 0.19	*p* = 0.162, η^2^ = 0.134	*p* = 0.413, η^2^ = 0.048
**Lean body mass (kg)**	54.57 ± 4.31	55.40 ± 4.69	55.87 ± 10.02	56.68 ± 10.34	*p* < 0.001, η^2^ = 0.705	0.82 (0.59, 1.05), ES = 0.10	*p* = 0.748, η^2^ = 0.008	*p* = 0.975, η^2^ = 0.000
**Leg lean mass (kg)**	18.41 ± 1.57	19.25 ± 1.87	19.29 ± 2.78	20.17 ± 3.08	*p* < 0.001, η^2^ = 0.723	0.86 (0.63, 1.08), ES = 0.35	*p* = 0.463, η^2^ = 0.039	*p* = 0.904, η^2^ = 0.001
**Trunk lean mass (kg)**	29.21 ± 3.39	29.26 ± 3.33	29.76 ± 6.47	29.72 ± 6.47	*p* = 0.756, η^2^ = 0.007	0.00 (−0.08, 0.08), ES = 0.00	*p* = 0.896, η^2^ = 0.001	*p* = 0.205, η^2^ = 0.112
**Arm lean mass (kg)**	6.95 ± 0.89	6.88 ± 0.93	6.81 ± 0.95	6.80 ± 0.96	*p* = 0.633, η^2^ = 0.017	−0.04 (−0.17, 0.09), ES = 0.04	*p* = 0.807, η^2^ = 0.004	*p* = 0.748, η^2^ = 0.008
**Head mass (kg)**	5.24 ± 0.24	5.13 ± 0.38	5.21 ± 0.35	5.21 ± 0.32	*p* = 0.251, η^2^ = 0.093	−0.06 (−0.02, 0.14), ES = 0.17	*p* = 0.869, η^2^ = 0.002	*p* = 0.262, η^2^ = 0.089
**Total body BMC (kg)**	3.01 ± 0.30	2.96 ± 0.33	2.92 ± 0.33	2.91 ± 0.32	*p* = 0.409, η^2^ = 0.049	−0.03 (−0.05, 0.00), ES = 0.08	*p* = 0.661, η^2^ = 0.014	*p* = 0.529, η^2^ = 0.029

Values are means ± standard deviation. Bold font highlights statistically significant differences. 90% CI, 90% confidence interval; η^2^, partial eta squared; ES, effect size; BMC, bone mineral content; BMI, body mass index; WC, waist circumference.

**Table 4:** Changes in carbohydrate and fat oxidation indices before (pre) and after (post) the intervention for the two training groups (HIIT10 and HIIT60).

	HIIT10		HIIT60		Pre-Post Change			
	Pre	Post	Pre	Post	(90% CI)	Effects	*p*-Value; ES	η^2^
**PFO**	0.20	0.33	0.24	0.34	0.12	Time (pre vs. post)	**<0.001; ES: 1.65**	0.699
**(g/min)**	±0.05	±0.08	±0.06	±0.09	(0.08, 0.15)	Group	0.536	0.028
						Time × Group	0.525	0.030
**PFO**	3.72	6.11	4.23	6.19	2.18	Time (pre vs. post)	**<0.001; ES: 1.72**	0.670
**(mg/min**	±0.74	±1.24	±0.82	±1.92	(1.54, 2.82)	Group	0.54	0.027
**/kg LBM)**						Time × Group	0.597	0.020
**Fat_peak_**	33.2	36.6	34.1	38.6	3.9	Time (pre vs. post)	**0.039; ES: 0.68**	0.270
**(%VO_2peak_)**	±5.5	±7.1	±3.8	±6.5	(1.2, 6.7)	Group	0.547	0.026
						Time × Group	0.783	0.006
**Fat_peak_**	60.1	62.2	58.7	63.6	3.5	Time (pre vs. post)	**0.011; ES: 0.10**	0.383
**(%HR_peak_)**	±5.8	±5.3	±7.3	±4.4	(1.5, 5.5)	Group	0.989	<0.001
						Time × Group	0.263	0.089
**COP**	29.8	40.8	32.3	36.9	7.8	Time (pre vs. post)	**0.023; ES: 1.01**	0.363
**(%VO_2peak_)**	±6.2	±6.9	±6.2	±10.4	(3.2, 12.4)	Group	0.793	0.006
						Time × Group	0.301	0.089
**PCHO**	2.42	2.93	2.41	3.11	0.61	Time (pre vs. post)	**<0.001; ES = 1.03**	0.620
**(g/min)**	±0.45	±0.68	±0.62	±0.52	(0.32, 0.89)	Group	0.732	0.009
						Time × Group	0.466	0.038

Values are expressed as means ± standard deviation. Bold font highlights statistically significant differences. 90% CI, 90% confidence interval; η^2^, partial eta squared; ES, effect size; COP, crossover point; PCHO, peak carbohydrate oxidation rate; Fat_peak_, exercise intensity corresponding to peak fat oxidation; HR_peak_, peak heart rate; LBM, lean body mass; PFO, peak fat oxidation rate; VO_2peak_, peak oxygen uptake; W_peak_, peak power.

## Data Availability

The data presented in this study are available on request from the corresponding author.
